# Is there any role of staging laparoscopy in pancreatic adenocarcinoma?

**DOI:** 10.1186/s12957-023-02975-1

**Published:** 2023-05-17

**Authors:** Theresa Soni, Jaiveer Singh, Bharath Nagarajan, Priyadarshini Velmurugan, Sudharsanan Sundaramurthi

**Affiliations:** Department of Surgery, Jawaharlal Institute of Postgraduate Medical Education and Research (JIPMER), Puducherry, India

**Keywords:** Pancreatic adenocarcinoma, Staging laparoscopy, CA 19-9, Pancreatic surgery

## Abstract

This is a letter to the editor on a study by Jambor et al. on the role of staging laparoscopy in identifying occult and distant metastases in pancreatic adenocarcinoma patients. In this study, inclusion of staging laparoscopy as an adjunct to computed tomography resulted in an absolute risk reduction of 12.5% for non-therapeutic laparotomy. The study found no correlation between the presence of occult and distant metastases, and serum CA 19-9 level, tumour size or location, which was in significant contrast to a number of other studies. This was likely due to the smaller sample size of the study and restriction to a single high-volume referral centre. It is also noted that staging laparoscopy cannot detect vascular invasion, lymph node involvement and deep hepatic metastases. The sensitivity of peritoneal lavage cytology in detecting occult metastases is low as well. Inclusion of biomarkers like peritoneal lavage tumour DNA may improve sensitivity. Hence, even as this study adds to the evidence supporting staging laparoscopy, further studies on improving the sensitivity of staging laparoscopy are warranted.

Dear Editor,

Pancreatic cancer, which is predominantly pancreatic ductal adenocarcinoma (PDAC), is the seventh most common cancer cause of death worldwide [1, 2]. Computed tomography (CT) is the conventional imaging modality for tumour staging and determination of surgical resectability, with endoscopic ultrasound (EUS), magnetic resonance imaging (MRI) and positron emission tomography (PET) as complementary tools [3]. PDAC is categorised into upfront resectable, borderline resectable, locally advanced disease or metastatic disease, and patients are appropriately selected to undergo either surgical resection or neoadjuvant chemotherapy [1]. Although curative surgical resection is the only option to achieve a cure, only about 15–20% of all pancreatic carcinoma patients have upfront resectable or borderline resectable disease [2].

Staging laparoscopy can be used as an adjunct diagnostic modality in pancreatic cancer. About 20–50% of patients undergo a non-therapeutic laparotomy due to the finding of occult and distant metastases during surgery [2]. Staging laparoscopy can prevent an unnecessary laparotomy by identifying distant or occult metastases that may be missed on CT [1]. However, improvements in the resolution of CTs and other imaging modalities (EUS, MRI, etc.) challenge the role of staging laparoscopy. Given the availability of limited resources, it also remains to be seen if staging laparoscopy should be advised in all cases of potentially resectable disease [4].

We congratulate the authors Jambor et al. on their retrospective study on the role of staging laparoscopy in pancreatic adenocarcinoma and its effect on patients’ survival. A total of 155 patients were determined to have resectable disease by triple-phase CT scan with pancreatic protocol, and were included in the study. Of them, 62 patients (40%) had upfront resectable, 53 (34%) had borderline resectable and 40 (26%) had locally advanced disease. Median CA 19-9 value was found to be 125 kU/L. Staging laparoscopy determined unresectability in 24 (15.5%) of these patients: nine (15%) upfront resectable patients, five (9%) borderline resectable patients and ten (25%) locally advanced patients. Only 3% of patients who underwent laparotomy after a negative staging laparoscopy had an abandoned resection due to the presence of liver metastases, hence the absolute risk reduction was 12.5% [1].

Another study by Fong et al. found that staging laparoscopy prevented a laparotomy in 24.1% of patients, i.e. staging laparoscopy is a valuable investigation in pancreatic adenocarcinoma [4]. A study by Rosa et al. proposed that staging laparoscopy should be performed in patients with CT suggestive of resectable disease, and CA 19-9 ≥ 150 kU/L or tumour size >3 cm [5]. Karabicak et al. also found that increased tumour size and high CA 19-9 levels were independent risk factors for latent distant organ metastasis. Peritoneal metastasis was more likely in patients with a pancreas body or tail tumour and larger tumour size [6]. However, the study by Jambor et al. found no correlation between the presence of occult and distant metastases, and serum CA 19-9 level, tumour size or location. The *p*-value for serum CA 19-9 was however 0.064, which is very close to the threshold of 0.05. This could likely be because the study had a smaller sample size and was restricted to a single high-volume referral centre.

We conclude that there is enough evidence in literature to prove the beneficial role of staging laparoscopy in PDAC. It is to be noted that laparoscopy cannot detect vascular invasion, lymph node involvement and deep hepatic metastases, and hence must be used in addition to other imaging modalities [5]. Vascular invasion can be detected by multidetector CT with pancreatic protocol, which is the primary imaging modality to evaluate resectability. CT or MRI may be used to detect hepatic metastases, while PET/CT is useful in detecting lymph node involvement [3]. Peritoneal lavage cytology, done as a part of staging laparoscopy, also has low sensitivity in detecting occult metastases. A study by M. Suenaga et al. suggests that peritoneal lavage tumour DNA could be included as a biomarker in staging laparoscopy to diagnose peritoneal dissemination with higher sensitivity [7]. Larger, multi-institutional studies are recommended to improve the sensitivity of staging laparoscopy in determining treatment strategies for patients with pancreatic adenocarcinoma.

## Data Availability

Not applicable

## Abbreviations

PDAC: Pancreatic adenocarcinoma
CT: Computed tomography
EUS: Endoscopic ultrasound
MRI: Magnetic resonance imaging
PET: Positron emission tomography
